# SNHG17 drives malignant behaviors in astrocytoma by targeting miR-876-5p/ERLIN2 axis

**DOI:** 10.1186/s12885-020-07280-8

**Published:** 2020-09-03

**Authors:** Fengping Du, Qian Hou

**Affiliations:** Department of Neurology, the Second Hospital of Heibei Medical University, No. 215 West Heping Road, Shijiazhuang, 050000 Hebei China

**Keywords:** SNHG17, miR-876-5p, ERLIN2, Astrocytoma

## Abstract

**Background:**

Astrocytoma is a common tumor type in primary central nervous system and has a high death rate around the world. Aberrant expression of long non-coding RNAs (lncRNAs) has been introduced by emerging studies to result in the development of diverse cancers.

**Methods:**

RT-qPCR examined the expression of SNHG17, miR-876-5p and ERLIN2, and western blot evaluated ERLIN2 protein level. RNA pull down and luciferase reporter assays illustrated the relationships between SNHG17 and its downstream molecules.

**Results:**

SNHG17 was up-regulated in astrocytoma cells. Moreover, SNHG17 silence could repress the proliferation, migration and invasion of astrocytoma cells. Besides, miR-876-5p was selected out as a downstream molecule of SNHG17 in astrocytoma. ERLIN2 was determined to be targeted by miR-876-5p. ERLIN2 mRNA and protein levels were lessened by miR-876-5p overexpression and SNHG17 silence. Additionally, miR-876-5p overexpression decelerated the biological processes of astrocytoma cells, so did ERLIN2 knockdown. More importantly, the impacts of SNHG17 down-regulation on the malignant behaviors of astrocytoma cells were counteracted by overexpressed ERLIN2 or inhibited miR-876-5p.

**Conclusions:**

SNHG17 could induce the progression of astrocytoma by sponging miR-876-5p to elevate the expression of ERLIN2. This study indicated that SNHG17 has a high potential to be a therapeutic target for astrocytoma.

## Background

Astrocytoma, derived from astrocytes, is a commonplace brain tumor with high degree of malignancy [1]. Astrocytoma accounts for 13–26% of intracranial tumors and 21.2–51.6% of gliomas [2]. Currently, the main method for treating astrocytoma is surgery and the next ones are radiotherapy and chemotherapy [3]. However, the whole prognosis of astrocytoma is still not optimistic [4]. Thus, it is so imperative to find a novel way to improve the outcomes of treatment.

Long non-coding RNAs (lncRNAs) are a kind of non-coding RNAs with no or limited protein coding abilities and more than 200 nt in length [5]. Recent reporters declared that lncRNAs have significant functions in the initiation and development of multiple cancers, embracing astrocytoma. For example, SOX2OT was suggested to contribute to the malignancy of astrocytoma by overexpressing miR-194-5p and miR-122 [6]. H19 was reported to have an oncogenic function in astrocytoma cells [7]. SNHG17 is a novel lncRNA whose detailed role has not been explained in astrocytoma.

Competing endogenous RNA (ceRNA) network has gained more and more attention from increasing essays. In this network, lncRNAs competitively interact with miRNAs to liberate mRNAs from binding with such miRNAs, so that these mRNAs could code into proteins to exert corresponding functions [8]. In this study, we intended to focus on the role of SNHG17 in ceRNA network in astrocytoma cells. MiRNAs were found to play crucial parts in regulating the development of diseases including gliomas. For instance, miR-520d-5p and miR-520d-3p were associated with down-regulation of SIPP Alpha protein in astrocytoma cells [9]. MiR-15a and miR-24-1 were regarded as prognostic biomarkers for pilocytic astrocytoma [10]. MiR-218-2 was an oncogene in glioblastoma cells and accelerated drug resistance via targeting CDC27 [11]. MiR-876-5p was described as a tumor inhibitor in the psoriasis [12]. In hepatocellular carcinoma, miR-876-5p could repress the progression via reducing DNMT3A expression [13]. Nevertheless, the function of miR-876-5p has not been elucidated in astrocytoma.

In this study, we analyzed the role of SNHG17 in astrocytoma cells. Moreover, how SNHG17 exerted its function in astrocytoma by modulating its downstream targets was investigated in ceRNA network. Finally, rescue assays were applied to illustrate the function of SNHG17/miR-876-5p/ERLIN2 axis in astrocytoma cells.

## Methods

### Cell culture

Human astrocytoma cell lines (LN-215, ADF, U138 and A-382) and human astrocytes (NHA), from ATCC cell bank (Manassas, VA), were cultured under 37 °C and 5% CO_2_. Cell samples were all cultured in DMEM, along with 10% FBS and 1% Penicillin-Streptomycin Solution (all; Invitrogen, Carlsbad, CA). All cells were authenticated via STR profiling and tested for mycoplasma contamination before use. Besides, the NCBI database confirmed no any contamination of these cell lines.

### RNA extraction and RT-qPCR

Total RNAs from cultured cell samples were extracted using TRIzol method (Invitrogen), the cDNA was then obtained after reverse transcription. SYBR R Premix Ex TaqTM II from Takara Bio (Shiga, Japan) was applied for RT-qPCR to assess gene expression, followed by the 2^-ΔΔCt^ method for calculation. GAPDH or U6 served as the internal references. The experiment was repeated in triplicate for three times.

### Transfection

LN-215 and U138 cells were transfected with the designed shRNAs and control-shRNAs (Genepharma Company, Shanghai, China) for SNHG17 and ERLIN2 employing the Lipofectamine 2000 (Invitrogen). Moreover, the miR-876-5p mimics/inhibitor and NC mimics/inhibitor, as well as the pcDNA3.1/ERLIN2 and NC-pcDNA3.1, were also designed by Genepharma. The sequences of mimics/inhibitors were as below: miR-876-5p mimics: 5′-UGGAUUUCUUUGUGAAUCACCA-3′; NC mimics: 5′-UAAUGUUCUCCUCUUGAUGAAG-3′; miR-876-5p inhibitor: 5′-UGGUGAUUCACAAAGAAAUCCA-3′; NC inhibitor: 5′-CAUCAACUGUAGAUAGGAAUCA-3′.

### EdU staining assay

The proliferation capacity of LN-215 and U138 cells was measured by EdU incorporation assay kit (Ribobio, Guangzhou, China). 100 μL of EdU medium diluent was added to each well (96-well plate) after cell transfection. Cells were fixed with 4% paraformaldehyde and permeated with 0.5% Troxin X-100. Following cell nucleus was stained by DAPI, cells were observed under fluorescence microscope. The experiment was repeated at least three times.

### Cell counting kit-8 (CCK8)

After transfection, cells were collected and inoculated in 96-well plates (10^4^ per well). For testing cell activity, 10 μL of CCK-8 reagent was added into each well to incubate cells at 37 °C for 2 h. Then the absorbance of each well was measured using a microplate reader at 450 nm wavelength. The experiment was repeated at least three times.

### Flow cytometry assay

Apoptosis analysis was performed using Annexin-V fluorescein isothiocyanate (FITC)/propidium iodide (PI) double-staining kit (Abcam, Cambridge, UK) according to the instructions of manufacturer. Then the analysis of apoptotic cells was performed using BD FACSCalibur flow cytometry (BD Biosciences, San Jose, CA). The experiment was repeated at least three times.

### TUNEL staining assay

After transfection, cells were fixed by 4% formaldehyde at room temperature for 1 h, and then permeated on ice with 0.1% Triton x-100 for 2 min. Then cells were incubated with TUNEL reaction mixture after washing by PBS. The nucleus was stained with DAPI and then cells were observed under an inverted fluorescence microscope. The experiment was repeated at least three times.

### Sphere formation assay

Cells in sphere medium were inoculated into 96-well ultra-low attachment plates (Corning Inc., New York, NY) at a density of 10 cells per well. After 7 days, sphere cells were counted manually. The experiment was repeated at least three times.

### Transwell assay

Cells were suspended in serum-free DMEM and inoculated into Transwell upper compartment (Corning, Corning, NY) with matrigel for cell invasion, without matrigel for cell migration. Lower chamber was supplemented with complete medium. 24 h later, the migratory and invasive cells were observed under microscope after fixing by 4% formaldehyde and staining with 0.5% crystal violet. The experiment was repeated at least three times.

### Western blot

Total proteins were obtained after cells lysed by RIPA lysis and then separated via 12% SDS-PAGE, followed by shifting to PVDF membranes. Afterwards, the membranes were blocked by 5% nonfat milk and then incubated overnight at 4 °C with primary antibodies including rabbit monoclonal antibody against ERLIN2 (ab128924, 1:10000 dilution; predicted at 38 kDa but detected at 43 kDa; validated in HEK-293 T and HepG2 cells) and the Rabbit monoclonal antibody to GAPDH (ab128915, 1:20000; predicted at 36 kDa but detected at 35 kDa; validated in HepG2 cells). Following washing by TBST for three times, membranes were processed with the HRP-labelled goat anti-rabbit IgG antibody (ab205718, 1:50000 dilution; validated in human liver tissue lysate and HeLa cells) at room temperature for 2 h. All antibodies were acquired from Abcam (Cambridge, MA). The bands were observed by using the enhanced chemiluminescence system (ECL; Santa Cruz Biotechnology, Santa Cruz, CA). The experiment was repeated in triplicate for three times.

### Subcellular fractionation

Subcellular fractionation was achieved based on the instruction of PARIS™ kit (Thermo Fisher Scientific, Waltham, Massachusetts). GAPDH was used as the cytoplasmic reference and U6 as the nuclear reference. Expression level of SNHG17 in indicated fractions was assayed by RT-qPCR. The experiment was repeated in triplicate for three times.

### Fish

The specific SNHG17-FISH probe was synthesized by RiboBio and then utilized as per the protocol. Cell nuclei were counterstained with Hoechst. After that, cells were observed by scanning confocal microscope. The experiment was repeated at least three times.

### RNA pull down assay

The extracts from U138 and LN-215 cells were prepared and mixed with the biotinylated RNAs and beads. After 1 h, RNA enrichment in pull-downs was assayed by RT-qPCR. Relevant sequences were: miR-876-5p-WT: 5′-UGGAUUUCUUUGUGAAUCACCA-3′; miR-876-5p-Mut: 5′-UCCUAAAGUUUGUGAAUCACCA-3′. A nonsense sequence was applied as the negative control. The experiment was repeated at least three times.

### RNA immunoprecipitation (RIP)

U138 and LN-215 cells (1 × 10^7^) were lysed in the RIP lysis buffer and then reaped for culturing with the specific antibody to human Ago2. Normal mouse IgG antibody acted as the negative control. The precipitated RNAs were detected using RT-qPCR. The experiment was repeated at least three times.

### Luciferase reporter assay

Full-length of SNHG17 and ERLIN2 3’UTR sequences with wild-type or mutant miR-876-5p binding sites were inserted into pmirGLO vectors (Promega, Madison, WI), termed SNHG17-WT/Mut and ERLIN2-WT/Mut. After co-transfection of miR-876-5p mimics or NC mimics with above indicated reporters, the luciferase activity of each group was analyzed with luciferase reporter assay system (Promega). The experiment was repeated at least three times.

### Statistical analyses

All experiments were repeated at least three times. Data were exhibited as the manner of mean ± standard deviation (SD). Statistical analysis was performed by Prism 5.0 software (GraphPad, San Diego, CA). After validating that data meet the assumptions of indicated tests, the significance of group difference was evaluated using two-sided Student’s t-test and one-way ANOVA, as appropriate. The value of *p* < 0.05 (without adjustment for multiple comparisons) was set as the threshold of statistical significance in differences.

## Results

### SNHG17 was up-regulated and promoted malignant processes in astrocytoma cells

**Fig. 1:**
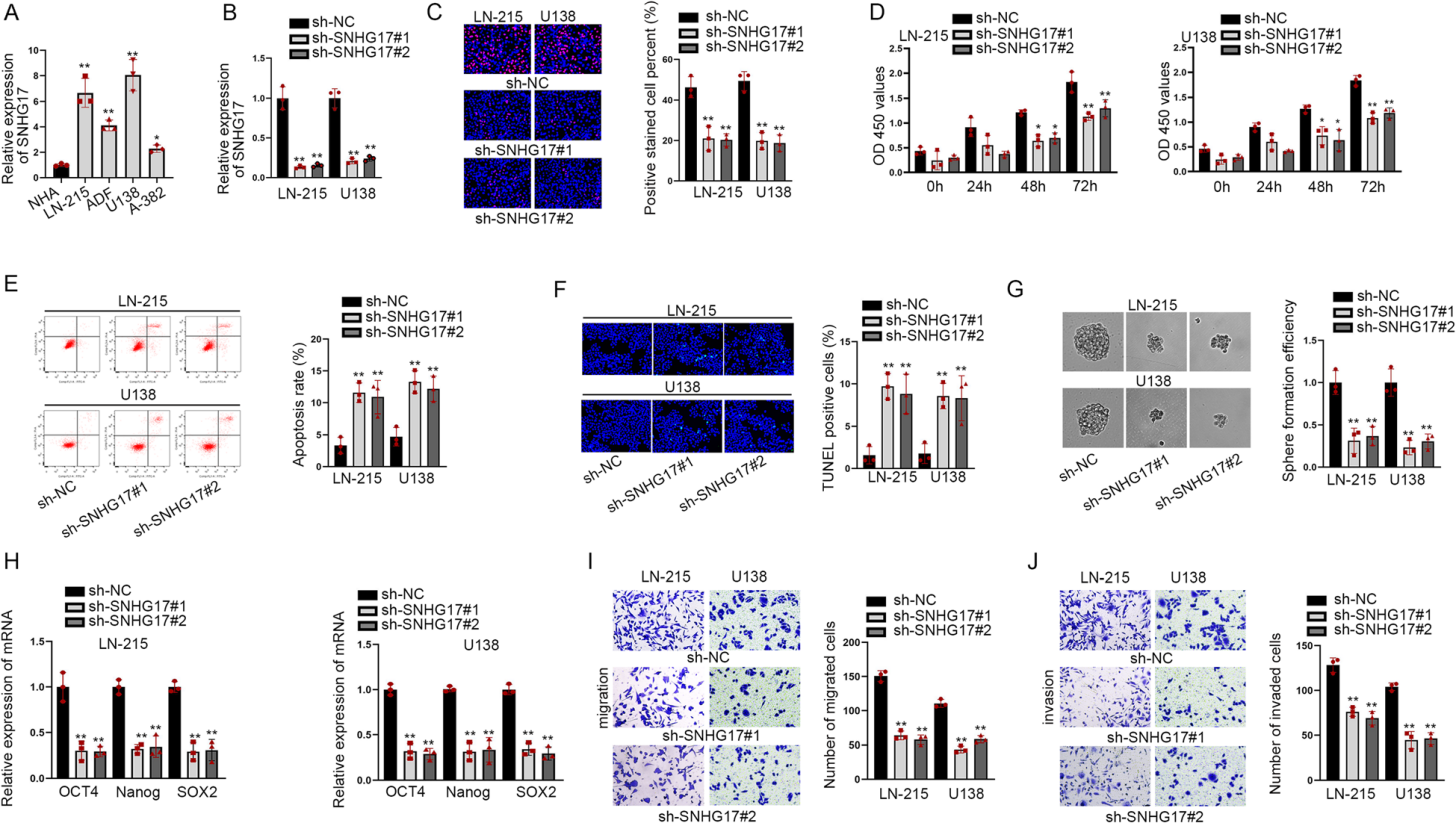
SNHG17 was up-regulated in astrocytoma cell lines and promoted the process of astrocytoma cells (**a**) SNHG17 expression was detected in astrocytoma cell lines (LN-215, ADF, U138 and A-382) and human astrocytes (NHA). **b** SNHG17 expression was examined in cells transfected with sh-SNHG17#1/2. **c**-**d** EdU assays and CCK8 assessed proliferation in sh-SNHG17#1/2 and sh-NC group. **e**-**f** Flow cytometry analysis and TUNEL appraised apoptosis in different groups. **g**-**h** Sphere formation efficiency was assessed by sphere formation assay and the expression of stemness-associated markers was measured by RT-qPCR. **i**-**j** Transwell assays evaluated cell migration and invasion in different groups. ^*^P < 0.05, ^**^P < 0.01

To understand the role of SNHG17 in astrocytoma, we first employed RT-qPCR to detect its expression. Data revealed that SNHG17 expressed at a high level in astrocytoma cells (LN-215, ADF, U138 and A-382) compared with normal astrocytes (NHA) (Fig. 1a). Considering the higher expression of SNHG17 in LN-215 and U138 cells, they were used in further functional assays. Next, LN-215 and U138 cells were transfected with sh-SNHG17#1/2 to decrease the expression of SNHG17 (Fig. 1b). Then EdU and CCK8 assays indicated that the proliferation of astrocytoma cells was restrained by knockdown of SNHG17 (Fig. 1c-d). On the contrary, astrocytoma cell apoptosis rate was augmented after down-regulating SNHG17 (Fig. 1e-f). Meanwhile, sphere formation efficiency of these two cells was also reduced dramatically by SNHG17 silence (Fig. 1g). Consistently, the expressions of stemness-related markers, including OCT4, Nanog and SOX2, were diminished due to SNHG17 knockdown (Fig. 1h). Likewise, the migration and invasion abilities of astrocytoma cells were lessened by down-regulated SNHG17 (Fig. 1i-j). In summary, SNHG17 had a powerful expression and promoted cell proliferation, migration, invasion and stemness in astrocytoma.

### MiR-876-5p suppressed cell proliferation and motility in astrocytoma

**Fig. 2:**
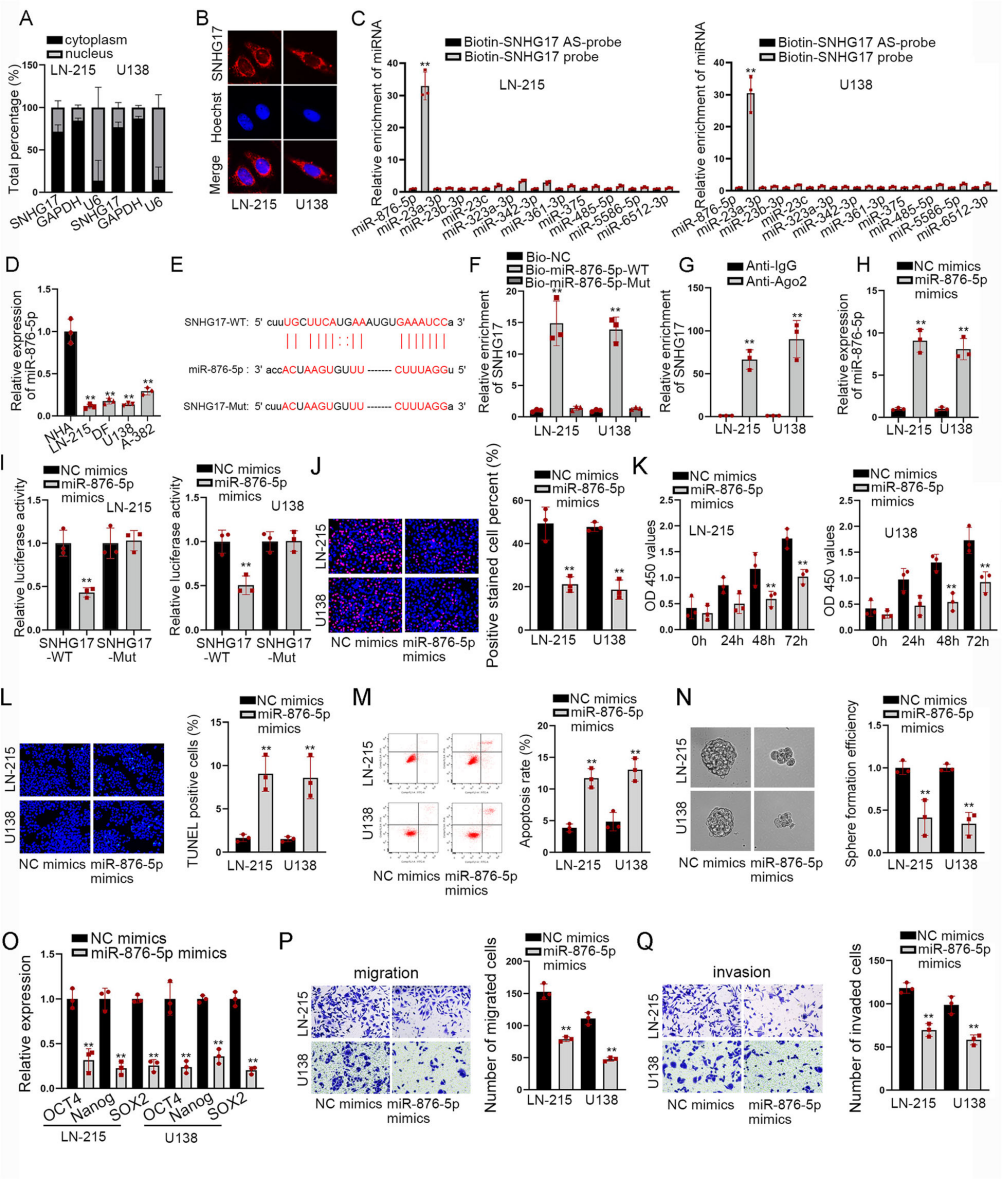
MiR-876-5p suppressed proliferation and motility in astrocytoma cells (**a**-**b**) Nuclear cytoplasm fractionation and FISH were built to judge the subcellular place of SNHG17 in U138 and LN-215 cells. **c** RNA pull down was used to select out miRNAs could bind to SNHG17. **d** MiR-876-5p expression was tested in astrocytoma cell lines. **e** The binding sites between SNHG17 and miR-876-5p were predicted by starBase. **f** RNA pull down manifested that miR-876-5p could bind to SNHG17. **g** RIP verified that SNHG17 could bind to Ago2. **h** MiR-876-5p expression was detected in astrocytoma cells. **i** Luciferase reporter assays illustrated that SNHG17 could bind to miR-876-5p in both LN-215 and U138 cells. **j**-**k** Proliferative capacities were examined in EdU and CCK8 in cells with NC mimics and miR-876-5p mimics. **l**-**m** Cell apoptosis was evaluated in different groups by TUNEL assay and flow cytometry analysis. **n**-**o** Stemness of cells in different groups was probed via sphere formation assay and RT-qPCR analysis of related genes. **p**-**q** Transwell assay examined migration and invasion. ^**^P < 0.01

The investigation on the regulatory mechanism of SNHG17 in astrocytoma begins with exploring its subcellular place. Data of nuclear cytoplasm fractionation and FISH assays exhibited that SNHG17 was primarily distributed in the cytoplasm (Fig. 2a-b), which unveiled the possibility of SNHG17 to participate in a ceRNA network in astrocytoma. By searching starBase v2.0 (http://starbase.sysu.edu.cn; School of Life Science, Sun Yat-sen University, China.), we found that there were 11 miRNAs with potential binding to SNHG17. The outcomes of RNA pull down assays presented that among these miRNAs, miR-876-5p was enriched mostly by biotin-SNHG17 probe (Fig. 2c). Data of RT-qPCR manifested that miR-876-5p had a low expression in astrocytoma cells (Fig. 2d). Then, the binding sites between SNHG17 and miR-876-5p were predicted by starBase (Fig. 2e). Subsequent results of RNA pull down assay manifested that SNHG17 could be pulled down by biotinylated miR-876-5p-WT but not by biotinylated miR-876-5p-Mut (Fig. 2f). The results of RIP assays displayed that SNHG17 was enriched by Ago2 antibody in both LN-215 and U138 cells (Fig. 2g). To get high expression of miR-876-5p, miR-876-5p mimics were transfected into cells and RT-qPCR proved the indeed overexpression of miR-876-5p after transfection (Fig. 2h). Further, we discovered that the luciferase activity of SNHG17-WT was falling in groups with miR-876-5p mimics, while that of SNHG17-Mut was not affected (Fig. 2i). Thereafter, we analyzed the impacts of miR-876-5p on the function of astrocytoma cells. It was showcased that the proliferation of astrocytoma cells was cut down by overexpression of miR-876-5p (Fig. 2j-k), whereas the apoptosis rate was elevated by miR-876-5p up-regulation (Fig. 2l-m). At the same time, sphere formation efficiency and the levels of stemness-associated markers were dropped after elevating miR-876-5p expression (Fig. 2n-o). Similarly, there was a decline in the number of migrated and invaded astrocytoma cells in response to miR-876-5p upregulation (Fig. 2p-q). Taken together, miR-876-5p could bind to SNHG17 and repressed the malignant course of astrocytoma cells.

### ERLIN2 worked as the downstream target of miR-876-5p and contributed to the progression of astrocytoma

**Fig. 3:**
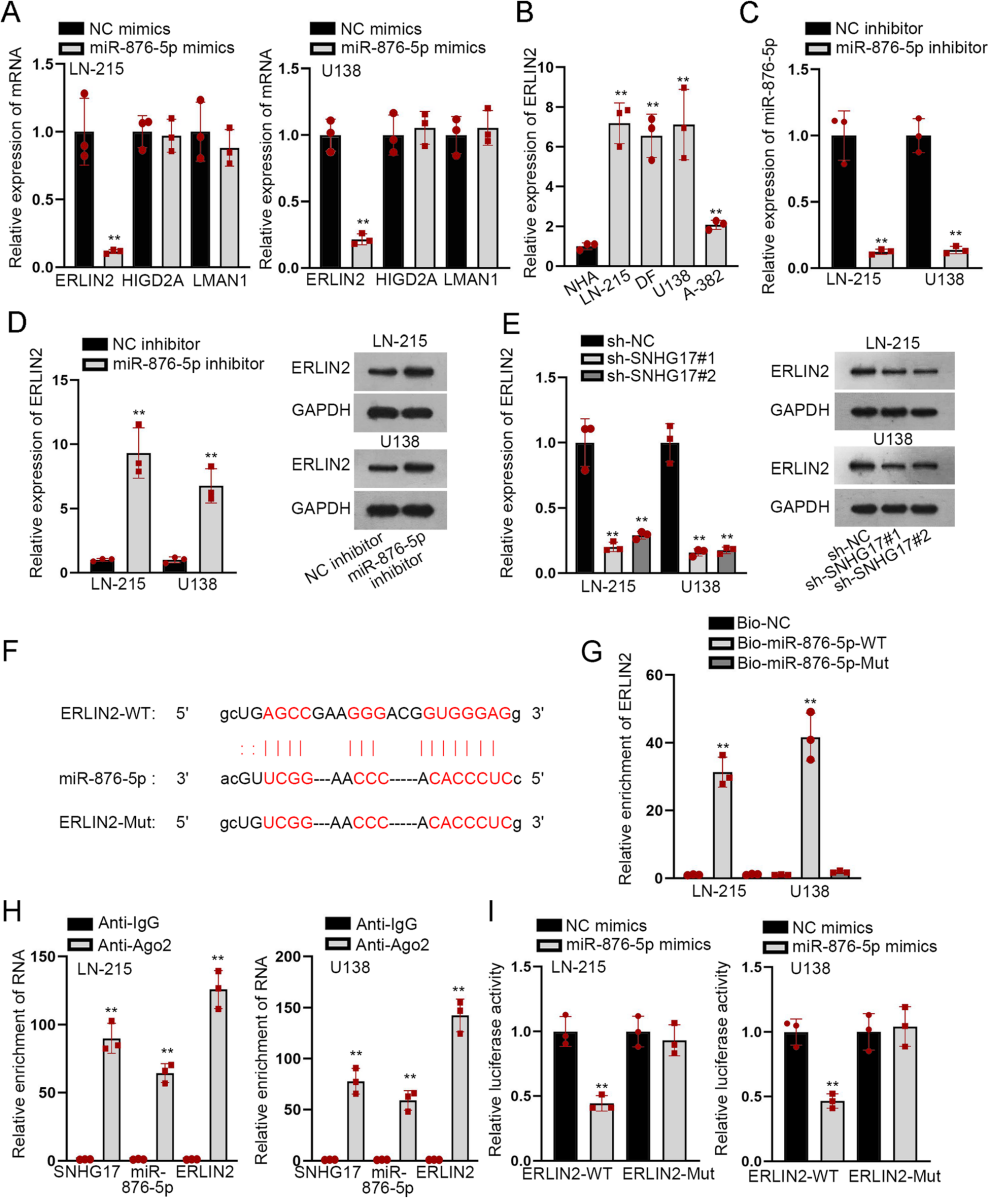
ERLIN2 worked as the downstream target of miR-876-5p and contributed to the progression of astrocytoma (**a**) RT-qPCR assays were carried out to measure mRNAs expressions in cells transfected with miR-876-5p mimics. **b** ERLIN2 expression was evaluated in astrocytoma cell lines. **c** MiR-876-5p expression was examined in cells received miR-876-5p inhibitor. **d**-**e** ERLIN2 expression was assessed in cells transfected with miR-876-5p inhibitor and sh-SNHG17#1/2. **f** The binding sequences between ERLIN2 and miR-876-5p were predicted by starBase. **g** RNA pull down certified miR-876-5p bound to ERLIN2. **h** RIP validated that SNHG17, miR-876-5p and ERLIN2 coexisted in RNA induced silencing complexes (RISCs). **i** Luciferase reporter assays verified that ERLIN2 bound to miR-876-5p. ^**^P < 0.01

By employing starBase, ERLIN2, HIGD2A and LMN1 were predicted as probable targets of miR-876-5p in line with following conditions: strict stringency> = 5, high stringency> = 3, Pan Cancer 4 types. However, only the expression of ERLIN2 was decreased by miR-876-5p mimics, while no conspicuous change could be seen in that of others (Fig. 3a). RT-qPCR revealed that ERLIN2 was up-regulated in astrocytoma cells compared to NHA (Fig. 3b). Then, we got lowered expression of miR-876-5p in LN-215 and U138 cells via transfecting with miR-876-5p inhibitor (Fig. 3c). As expected, we disclosed that ERLIN2 expression at both mRNA and protein levels was increased by miR-876-5p inhibition but descended after silencing SNHG17 (Fig. 3d-e). The binding sequence between ERLIN2 3’UTR and miR-876-5p was inferenced via starBase, as shown in Fig. 3f. RNA pull down experiments validated that miR-876-5p could bind to ERLIN2 (Fig. 3g). Meanwhile, RIP assays showed that SNHG17, ERLIN2 and miR-876-5p were all precipitated by Ago2 antibody (Fig. 3h). Luciferase reporter assays further illustrated the interaction between miR-876-5p and ERLIN2 (Fig. 3i).

Subsequently, we analyzed the effects of ERLIN2 on the biological behaviors of astrocytoma cells. First, we discovered the decreased expression of both ERLIN2 mRNA and protein in LN-215 and U138 cells after transfection with sh-ERLIN2#1/2 (Supplementary Fig. 1A). The following functional assays manifested that down-regulated ERLIN2 hindered astrocytoma cell proliferation by applying EdU and CCK8 assays (Supplementary Fig. 1B-C). Conversely, ERLIN2 knockdown enhanced the apoptosis of astrocytoma cells as detected by flow cytometry analysis and TUNEL assay (Supplementary Fig. 1D-E). Furthermore, sphere formation efficiency was reduced and the levels of markers related to stemness were also lowered under ERLIN2 down-regulation (Supplementary Fig. 1F-G). Likewise, the migratory and invasion capacities of astrocytoma cells were repressed by ERLIN2 silence (Supplementary Fig. 1H-I). In a word, ERLIN2 targeted by miR-876-5p facilitated the malignancy in astrocytoma.

### SNHG17 boosted the malignant phenotypes of astrocytoma cells through targeting miR-876-5p/ERLIN2 signaling

**Fig. 4:**
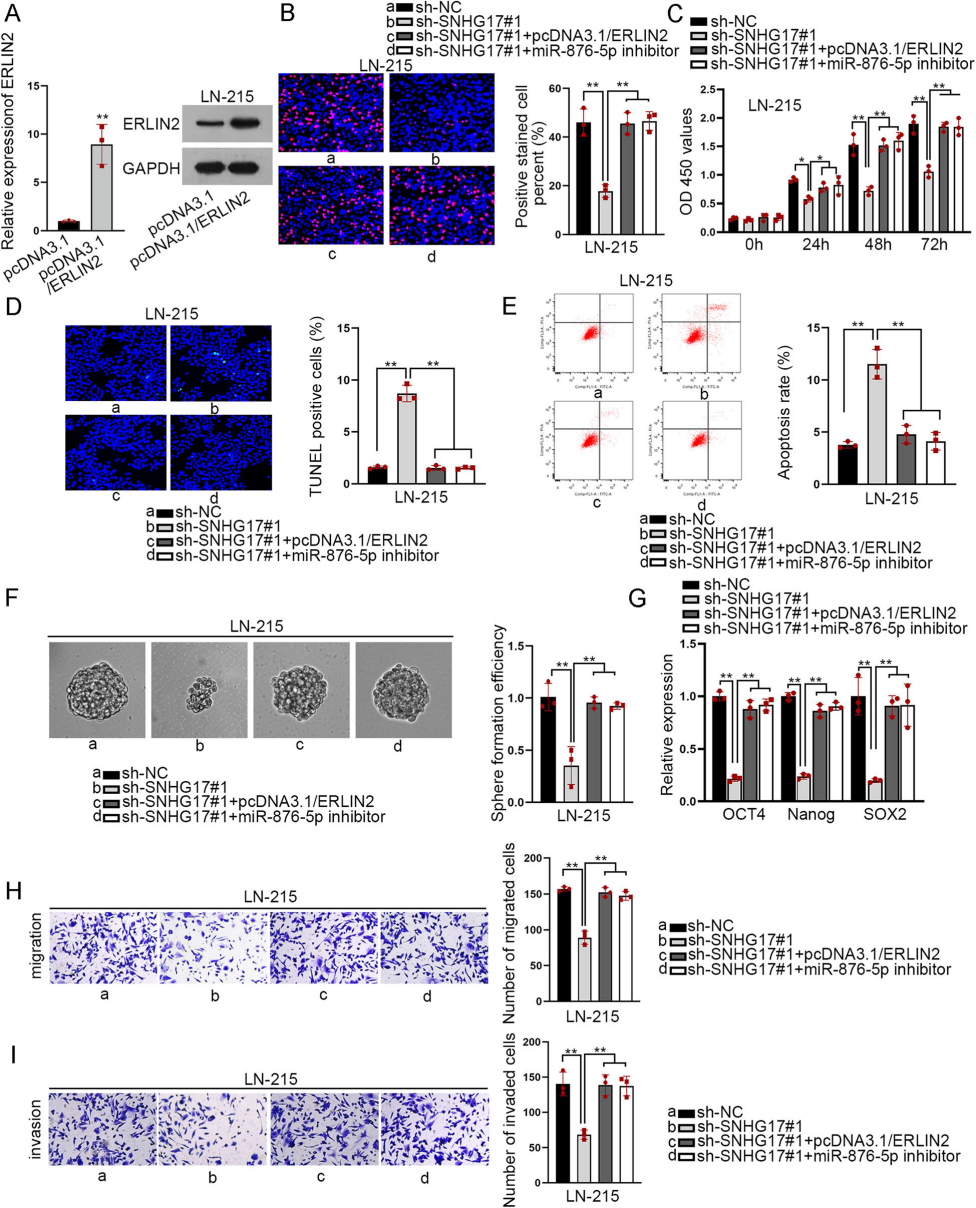
SNHG17 boosted the growth of astrocytoma through targeting miR-876-5p/ERLIN2 (**a**) RT-qPCR assays were carried out to measure ERLIN2 expression transfected with pcDNA3.1/ERLIN2. **b**-**i** Rescue assays appraised proliferation, apoptosis, migration and invasion as well as sphere efficiency in sh-NC, sh-SNHG17#1 and pcDNA3.1/ERLIN2. ^**^P < 0.01

Finally, we implemented rescue assays to validate the effectiveness of SNHG17/miR-876-5p/ERLIN2 axis in astrocytoma cells. To begin with, pcDNA3.1/ERLIN2 was transfected into LN-215 cells to increase the expression of ERLIN2 at both mRNA and protein levels (Fig. 4a). The descending trend of cell proliferation mediated by suppressed SNHG17 was reversed by overexpression of ERLIN2 or down-regulation of miR-876-5p (Fig. 4b-c). The ascending apoptosis induced by SNHG17 silence was counteracted by down-regulated miR-876-5p or up-regulated ERLIN2 (Fig. 4d-e). The falling tendencies of sphere formation efficiency and expression of stemness-related markers imposed by SNHG17 knockdown were restored under ERLIN2 overexpression or miR-876-5p inhibition (Fig. 4f-g). In addition, the hindered cell migration and invasion caused by SNHG17 down-regulation were offset by miR-876-5p depletion or ERLIN2 overexpression (Fig. 4h-i). Moreover, we also found that depleted SNHG17 markedly enhanced the sensitivity of LN-215 cells to temozolomide (TMZ), while miR-876-5p inhibition or ERLIN2 upregulation could reverse such sensitization effect (Supplementary Fig. 2A), indicating that SNHG17/miR-876-5p/ERLIN2 pathway might also work in the formation of TMZ-resistance in astrocytoma. Altogether, SNHG17 could facilitate malignancy in astrocytoma through targeting miR-876-5p/ERLIN2 axis.

## Discussion

Astrocytoma is a kind of brain tumor with high malignancy. The current treatment has limited impacts on astrocytoma and the recurrence rate is high in patients with astrocytoma. With the advancement of science and technology, target therapy gets more and more attention. LncRNAs have been demonstrated to exert vital functions in regulating the development of diseases including glioma. For example, UCA1 accelerated the growth of glioma cells by decreasing miR-182 to target iASPP [14]. NEAT1 facilitated cell migration and invasion in glioma by regulating SOX2 via sponging miR-132 [15]. In this study, we found that SNHG17 was up-regulated in astrocytoma cells. Besides, down-regulation of SNHG17 could hamper proliferative, migratory and invaded abilities as well as sphere formation efficiency in astrocytoma cells. Previous studies validated that SNHG17 served as an oncogene in gastric cancer [16] and melanoma [17]. Our finding was consistent with these previous findings.

Mounting papers studied the role of ceRNA network in the development of diverse diseases. This network claimed that lncRNAs could serve as sponges of miRNAs. In this study, we confirmed that SNHG17 was mainly distributed in the cytoplasm of astrocytoma cells. Additionally, we disclosed that SNHG17 had the capacities to bind to miRNAs in astrocytoma cells. Previously, miR-876-5p was reported to inhibit the progression of breast cancer by targeting TFAP2A [18]. The former study manifested that miR-876-5p regulated cell invasion and metastasis in head and neck squamous cell carcinoma via targeting vimentin [19]. Herein, miR-876-5p was revealed to be down-regulated in astrocytoma cells. Furthermore, the functional assay verified that miR-876-5p had inhibitory effects on the malignancy in astrocytoma.

Subsequently, we selected out ERLIN2 as a target of miR-876-5p in astrocytoma cells. Current literatures have mainly unveiled the function of ERLIN2 in breast cancer (BC). For instance, ERLIN2 was reported to facilitate the survival of BC cells through regulating endoplasmic reticulum stress pathways [20]. ERLIN2 stabilized Cyclin B1 to facilitate cell cycle progression in BC [21]. Also, it was said that ERLIN2 was targeted by miR-410 in breast cancer to promote the progression [22]. Moreover, Wang, G., et al. suggested that ERLIN2 contributed to the proliferation of BC or hepatoma cells [23]. In this study, we detected that ERLIN2 was also up-regulated in astrocytoma cells. In addition, ERLIN2 accelerated the proliferation, migration, invasion and sphere formation ability in astrocytoma cells, which was similar to the results in breast cancer [20]. Moreover, the rescue assays revealed that ERLIN2 overexpression or down-regulation of miR-876-5p could reverse the impacts of SNHG17 silence on the function of astrocytoma cells. Interestingly, here we also found that SNHG17/miR-876-5p/ERLIN2 axis also contributed to the resistance of astrocytoma cells to temozolomide (TMZ), a routinely-used drug for treating patients with gliomas including astrocytoma [24]. However, more about the impact of this pathway in the chemoresistance of astrocytoma cells need to be verified by further studies in the future.

## Conclusions

In conclusion, our study found that SNHG17 promoted the proliferation, migration, invasion and stemness of astrocytoma cells via targeting miR-876-5p/ERLIN2 pathway. However, the detailed significance of SNHG17/miR-876-5p/ERLIN2 axis in chemoresistance of astrocytoma cells as well as in clinic practice needs to be further discussed and analyzed in the future.

## Supplementary information

**Additional file 1: Supplementary Figure 1.** (A) ERLIN2 expression at both mRNA and protein levels was measured by RT-qPCR and western blot in cells transfected with sh-NC or sh-ERLIN2#1/2. (B) The effect of ERLIN2 on astrocytoma cell proliferation was assessed by EdU assay. (C) CCK-8 assay detected the viability of astrocytoma cells under ERLIN2 silence. (D-E) The apoptosis of cells with or without ERLIN2 inhibition was determined by flow cytometry analysis and TUNEL assay. (F) Sphere formation assay evaluated the impact of ERLIN2 depletion on the stemness of astrocytoma cells. (G) RT-qPCR results of stemness-related genes under ERLIN2 suppression. (H-I) Cell migration and invasion under ERLIN2 silence or not were assessed by transwell assays. ^*^P < 0.05, ^**^P < 0.01.

**Additional file 2: Supplementary Figure 2.** (A) CCK-8 assay examined the sensitivity of LN-215 cells to TMZ when being transfected with sh-NC, sh-SNHG17#1, sh-SNHG17#1 + pcDNA3.1/ERLIN2, sh-SNHG17#1 + miR-876-5p inhibitor.

**Additional file 3: Supplementary file 1.** The original, uncropped western blot images for Figs. 3d, e, Fig. 4a and S1A.

## Data Availability

All data generated or analysed during this study are included in this published article and its supplementary information files.

## Abbreviations

lncRNAs: Long non-coding RNAs
ceRNA: Competing endogenous RNA
RT-qPCR: Real-time quantitative PCR
RIP: RNA immunoprecipitation
CCK8: Cell counting kit-8
PI: Propidium iodide
FITC: Fluorescein isothiocyanate
SD: Standard deviation
