# Supplementary figures and images for: SNHG17 drives malignant behaviors in astrocytoma by targeting miR-876-5p/ERLIN2 axis
Source: BMC Cancer. 2020 Sep 3;20:839. doi: 10.1186/s12885-020-07280-8 (PMC7469335; doi:10.1186/s12885-020-07280-8)

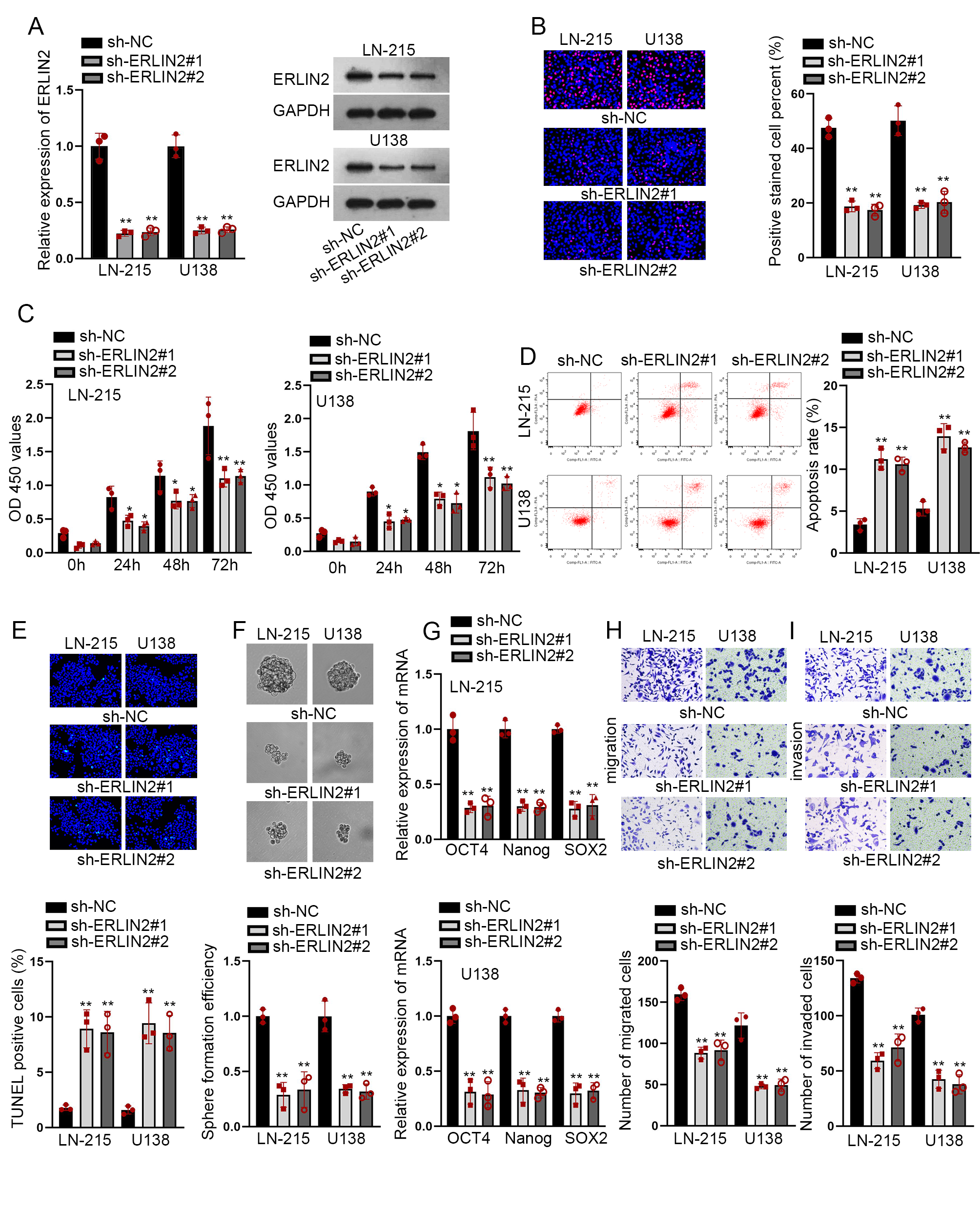

Supplement: Supplementary file 1 — Additional file 1: Supplementary Figure 1. (A) ERLIN2 expression at both mRNA and protein levels was measured by RT-qPCR and western blot in cells transfected with sh-NC or sh-ERLIN2#1/2. (B) The effect of ERLIN2 on astrocytoma cell proliferation was assessed by EdU assay. (C) CCK-8 assay detected the viability of astrocytoma cells under ERLIN2 silence. (D-E) The apoptosis of cells with or without ERLIN2 inhibition was determined by flow cytometry analysis and TUNEL assay. (F) Sphere formation assay evaluated the impact of ERLIN2 depletion on the stemness of astrocytoma cells. (G) RT-qPCR results of stemness-related genes under ERLIN2 suppression. (H-I) Cell migration and invasion under ERLIN2 silence or not were assessed by transwell assays. *P < 0.05, **P < 0.01. [file 12885_2020_7280_MOESM1_ESM.tif]

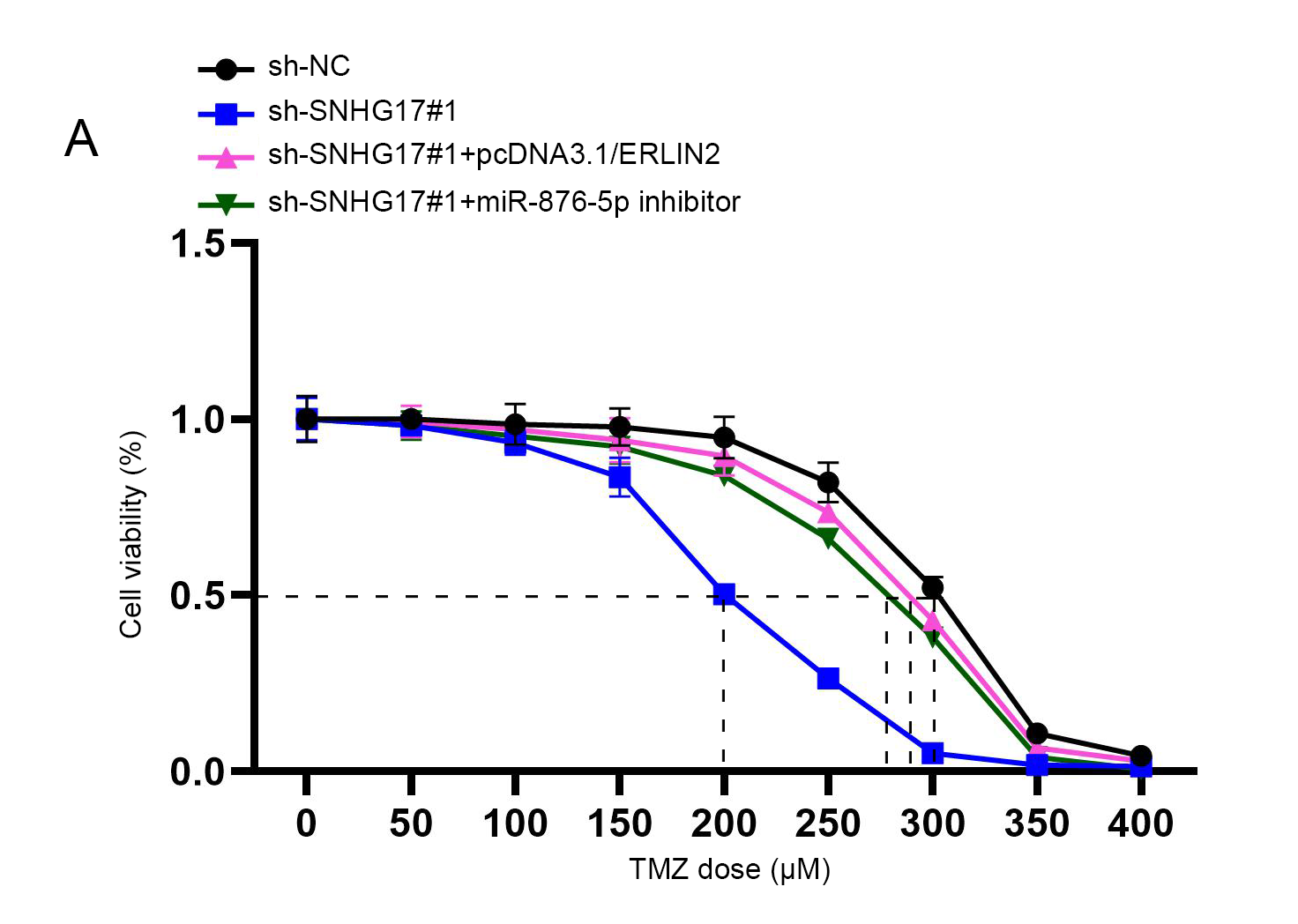

Supplement: Supplementary file 2 — Additional file 2: Supplementary Figure 2. (A) CCK-8 assay examined the sensitivity of LN-215 cells to TMZ when being transfected with sh-NC, sh-SNHG17#1, sh-SNHG17#1 + pcDNA3.1/ERLIN2, sh-SNHG17#1 + miR-876-5p inhibitor. [file 12885_2020_7280_MOESM2_ESM.tif]

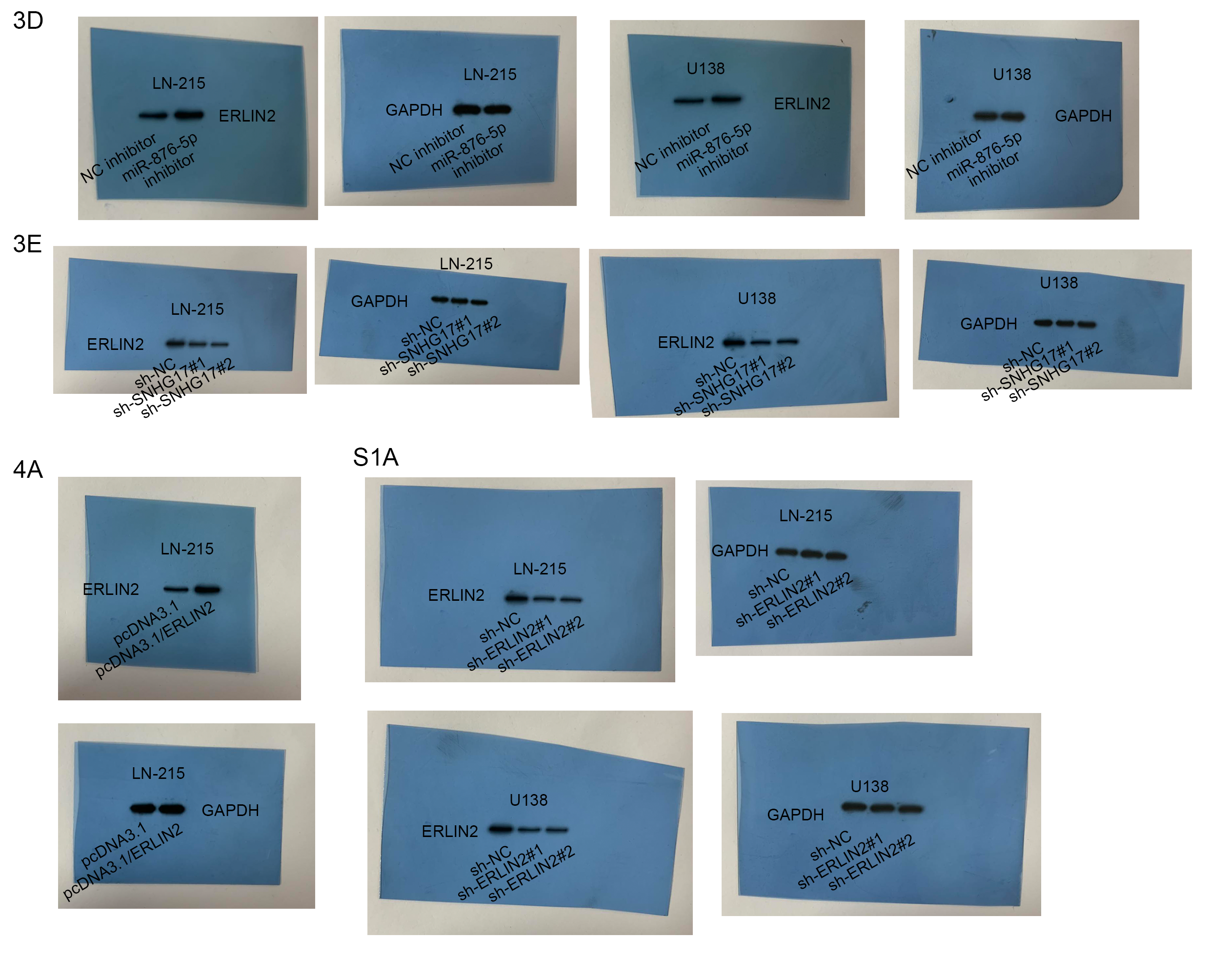

Supplement: Supplementary file 3 — Additional file 3: Supplementary file 1. The original, uncropped western blot images for Figs. 3d, e, Fig. 4a and S1A. [file 12885_2020_7280_MOESM3_ESM.tif]
